# An Atypical Presentation of Tuberculomas in an Immunocompetent Host

**DOI:** 10.1177/2324709618798407

**Published:** 2018-09-01

**Authors:** Frederick Venter, Arash Heidari, Kristine Galang, Macsen Viehweg

**Affiliations:** 1 University of California, Los Angeles, CA, USA; 2 Kern Medical Center, Bakersfield, CA, USA

**Keywords:** tuberculoma, *Mycobacterium*, tuberculosis, immunocompromised, immunocompetent, intracranial, lesion, meningitis, ring enhancing, caseating, granuloma, seizure

## Abstract

Tuberculomas are an intracranial form of tuberculosis that account for a third of intracranial lesions in endemic areas. If symptomatic, they usually present as meningitis in an immunocompromised host; however, in patients without signs of meningitis, clinical features are essentially indistinguishable from any other space-occupying lesion. We present a case of central nervous system tuberculosis in an immunocompetent host who presented with new-onset seizures.

## Introduction

*Mycobacterium tuberculosis* is the causative agent in tuberculosis (TB). It is highly aerobic and therefore affects primarily the respiratory system. In rare cases, where incidence of TB is high, *M tuberculosis* can spread through the circulatory system via the release of coalescing tubercules^1^ seeding the central nervous system (CNS) where they develop into tuberculomas usually preceded or accompanied by meningitis. This typically occurs in an immunocompromised host, although cases in immunocompetent patients have been documented. An even smaller portion of CNS tuberculomas are located deep enough in the brain parenchyma that they do not cause meningeal irritation. Deep tuberculomas can either remain asymptomatic or eventually cause headaches, induce seizures, and precipitate deficits caused by the mass effect of these space occupying lesions.

## Case Presentation

**Figure 1.**
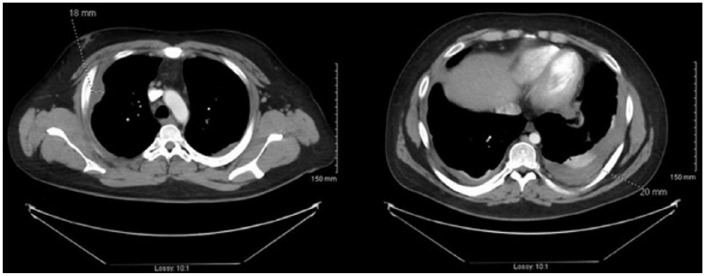
Computed tomography scan demonstrating pleural opacities and thickening.

A 24-year-old Hispanic male who previously worked as a nurse in Mexico presented to our facility 4 months prior as a self-referral. He had been suffering from recurrent bilateral pleural effusion and thickening for the past 2 years without any diagnosis (Figure 1).

**Figure 2.**
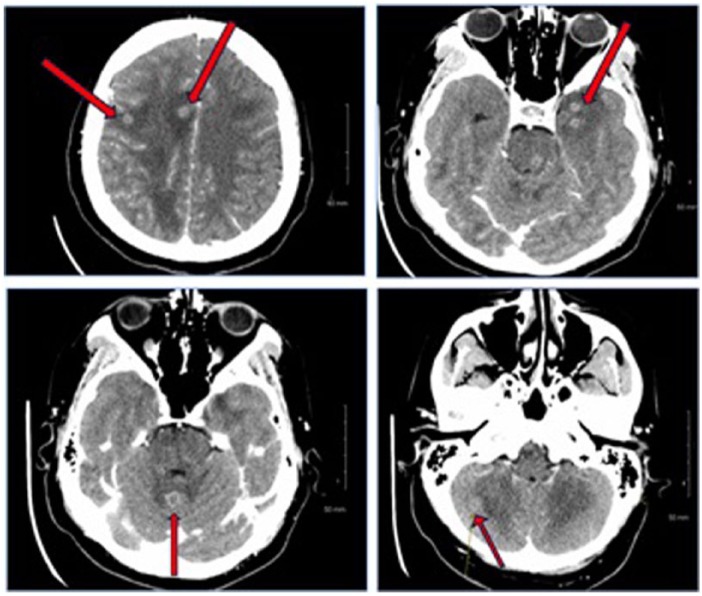
Computed tomography scan with numerous infratentorial and supratentorial tiny enhancing lesions.

**Figure 3.**
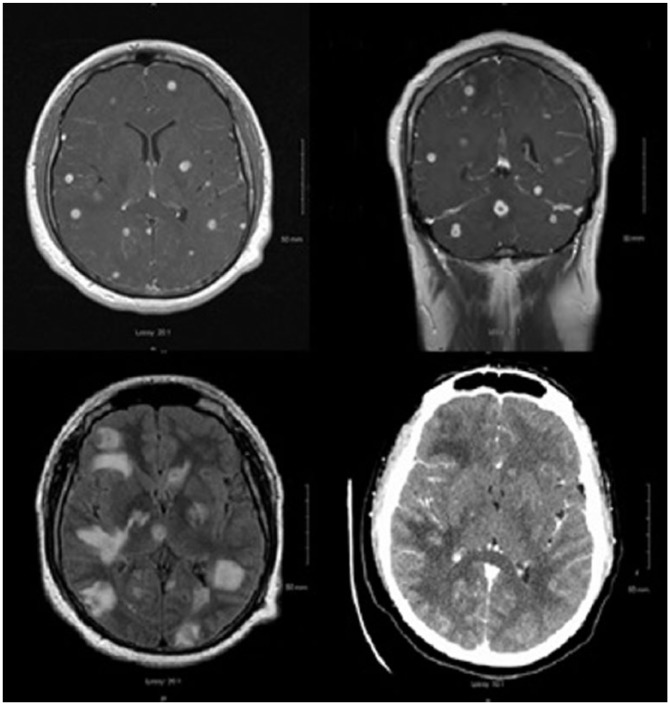
Magnetic resonance imaging (MRI) with numerous ring-enhancing lesions involving the infratentorial and supratentorial structures. Additional lesions were identified on the MRI examination compared with computed tomography.

During our initial workup, he was found to have a positive QuantiFERON-TB test but had negative sputum acid-fast bacilli (AFB) smear and culture and was discharged to follow-up in our pulmonary clinic. He was lost to follow-up and presented again, this time with new-onset headaches and seizures. Physical examination was significant for bitemporal visual deficits. A brain computed tomography (CT) and magnetic resonance imaging (MRI) revealed numerous infratentorial and supratentorial ring-enhancing brain lesions with vasogenic edema (Figures 2 and 3). At this point, our differentials were the following: neurocysticercosis versus tuberculomas versus toxoplasmosis versus lymphoma versus metastatic brain cancer. After the brain CT and MRI, and due to the patient not having any focal neurological deficits, reduced Glasgow Coma Scale, and abnormal respirations or papilledema, the decision was made to perform a lumbar puncture (LP) to rule in what we believed to be an infectious etiology. LP showed an opening pressure of 370 mm H_2_O, cerebrospinal fluid (CSF) white blood cell count of 8 × 10^3^/µL, and CSF glucose and protein were 50 mg/dL and 89 mm/dL, respectively, with a 55% lymphocyte predominance. The patient was also screened for HIV with an Ab/Ag (antibody/antigen) screen, which was nonreactive.

**Figure 4.**
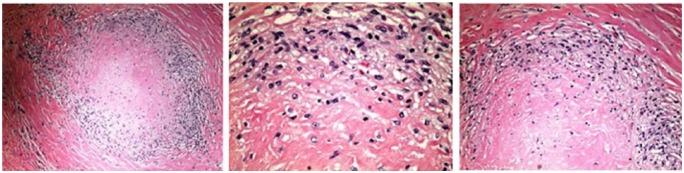
Caseating granuloma with hematoxylin-eosin stain at 10×, 20×, and 40×.

Due to a high index of suspicion for TB, he was empirically placed on 4 anti-TB medications and a steroid. A pleural biopsy was performed, which showed caseating granulomata pleural with negative AFB stain (Figure 4). Throughout hospitalization, he had 2 additional LPs to alleviate elevated intracranial pressure. Airborne isolation was cleared after 3 negative sputum AFBs, and he was discharged home with the same 4-drug regimen and a steroid taper dose. His biopsy grew *M tuberculosis* complex after 6 weeks in the laboratory and a report by the Public Health Services Department showed pansensitivity without any resistance. The patient’s drug regime consisted of isoniazid, rifampin, pyrazinamide, ethambutol, pyridoxine, and dexamethasone. All 4 anti-TB medications were given for 2 months with maintenance therapy consisting of isoniazid and rifampin for an additional 9 months. Dexamethasone was administered and tapered over a total of 8 weeks at 0.3 to 0.4 mg/kg/day for 2 weeks, 0.2 mg/kg/day for week 3, 0.1 mg/kg/day for week 4, and then 4 mg per day and tapered 1 mg off the daily dose each week. The patient’s symptoms rapidly improved with this drug regime, and repeat brain imaging a few weeks after initiation of medications revealed that some of the tuberculomas had already resolved.

## Discussion

Tuberculosis is a major health concern in developing countries where prevalence is high. When it spreads to the CNS, TB usually manifests as meningitis. It very rarely infects the brain parenchyma in immunocompetent individuals.^2^

TB can induce the formation of conglomerate granulomatous foci within the brain creating tuberculomas.^3^ This is achieved by the release of coalescing tubercles into the blood^1^ from the primary source, which is typically in the lungs. Tuberculomas within the brain sometimes remain asymptomatic. The cases that eventually become symptomatic are usually in immunocompromised hosts. Typical presentation is with signs and symptoms of meningitis; however, tuberculomas can be clinically silent when they do not cause any irritation to the meninges. Tuberculomas not causing meningeal symptoms most often present with new-onset seizures and headaches.^1^ They are typically seen in patients from high-risk countries. Tuberculomas can also present with hemiplegia and other signs of mass effect as well as raised intracranial pressure. They account for a third of intracranial lesions in endemic areas and are seen in 1% of TB cases.^4^ In patients without signs of meningitis, clinical features are essentially indistinguishable from any other space occupying lesion.^5^

On brain CT, early disease presents as low density or isodense lesions with a large amount of edema and little encapsulation but become encapsulated and hyperdense with peripheral ring enhancement as the disease advances.^1^

LPs are typically avoided due to fear of brain herniation, but when performed, they usually reveal normal and nonspecific results.^5^ Tuberculomas within the brain are usually treated pharmacologically and not with surgery as this may seed meningitis. Surgical intervention is indicated if the lesions are at risk of causing obstructive hydrocephalus.^1^ Diagnosis is usually made by evaluating the clinical presentation, epidemiology, and imaging studies and sometimes fine needle biopsy. Tuberculomas are often misdiagnosed as tumors or metastatic disease triggering health professionals to go hunting for cancer. Our case was officially diagnosed when a pleural biopsy we collected yielded *M tuberculosis* after 6 weeks of growth in the laboratory. Differentiating between tuberculomas and neurocysticercosis is difficult as they both present with similar signs, symptoms, and imaging studies.^1^

Typically, CSF analysis of CNS TB has lymphocytic pleocytosis, low glucose, and high protein, which was inconsistent with our patient who had a lower than expected lymphocytic count and a hyperglycorrachia.^4^

Treatment includes the standard 4-drug anti-TB drug regimen therapy, which should be initiated for strong clinical suspicion and not be delayed until bacteriologic confirmation has been obtained as this can take months.^5^ In our patient, despite our high clinical suspicion for TB, the pleural biopsy we obtained took 6 weeks to grow *M tuberculosis.*

## Conclusion

Tuberculomas are conglomerate granulomatous foci in the brain parenchyma typically presenting in an immunocompromised host as meningitis. Here we present an atypical case of tuberculomas in an immunocompetent host without meningitis who exhibited unusual cerebrospinal fluid laboratory results for CNS TB.
